# Amantadine Improves Delayed Neuropsychiatric Sequelae of Carbon Monoxide Poisoning: A Case Report

**DOI:** 10.3390/brainsci9110292

**Published:** 2019-10-25

**Authors:** Tomosuke Nakano, Toshiki Hasegawa, Dai Suzuki, Eishi Motomura, Motohiro Okada

**Affiliations:** Department of Neuropsychiatry, Mie University Graduate School of Medicine, 2-174, Edobashi, Tsu, Mie 514-8507, Japan; t-hasegawa@clin.medic.mie-u.ac.jp (T.H.); dsky@clin.medic.mie-u.ac.jp (D.S.); motomura@clin.medic.mie-u.ac.jp (E.M.); okadamot@clin.medic.mie-u.ac.jp (M.O.)

**Keywords:** amantadine, carbon monoxide poisoning, delayed neuropsychiatric sequelae, NMDA-R antagonist

## Abstract

Carbon monoxide (CO) poisoning causes severe brain damage, including delayed neuropsychiatric sequelae (DNS), which occur after a lucid interval following recovery from the insult of acute CO poisoning. We describe a 39-year-old male who developed DNS, including gait disturbance, trunk ataxia, and fecal/urine incontinence, after remission of acute CO poisoning. Furthermore, he showed confusion, with disorientation in terms of time and space. All symptoms, including cognitive impairment, were dramatically improved by amantadine monotherapy. The present case illustrates the possibility of amantadine treatment for cognitive impairment as well as Parkinsonism induced by CO poisoning.

## 1. Introduction

It is well known that carbon monoxide (CO) results in acute and chronic brain damage. Acute CO poisoning leads to delayed neuropsychiatric sequelae (DNS), which occur after a period of recovery. The characteristic symptoms are not specific and typically include cognitive impairment, excretory disorder, gait disturbance, and other neuropsychiatric manifestations [1]. In the clinical setting, hyperbaric oxygen is administered after CO exposure; however, there is no consensus regarding the effect of hyperbaric oxygen treatment in preventing DNS due to CO poisoning [2,3]. Thus, the treatment of DNS due to CO poisoning is not established. Here, we present a case with DNS due to CO poisoning, in which amantadine (AMA), a glutamine/N-methyl-D-aspartate receptor (NMDA-R) antagonist, improved cognitive impairment as well as Parkinsonism.

## 2. Case presentation

**Figure 1:**
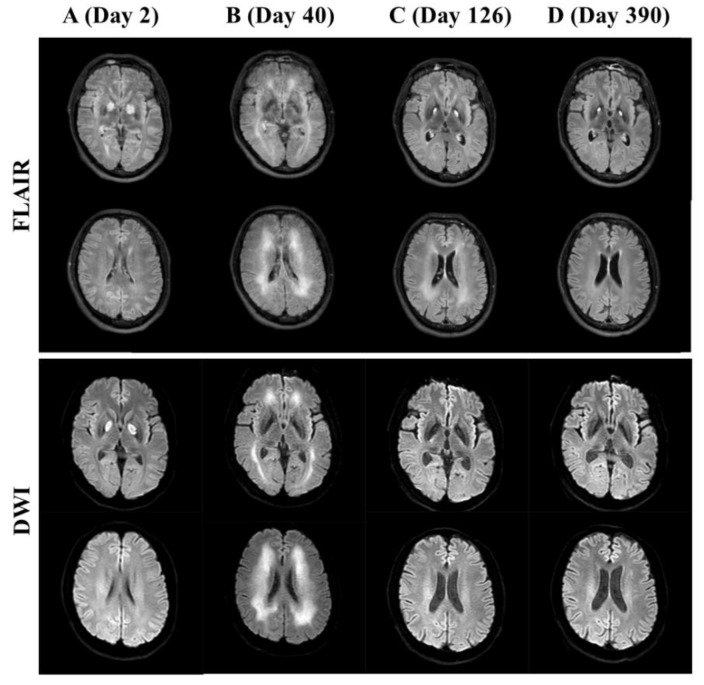
Serial brain magnetic resonance (MR) images. FLAIR, fluid-attenuated inversion recovery; DWI, diffusion-weighted imaging.

A 39-year-old male attempted suicide by CO poisoning from burning charcoal in a car and was admitted to the emergency department of Mie University Hospital for severe coma. A blood gas analysis exhibited pH 7.36, PaCO_2_ 34.0 (mmHg), PaO_2_ 327.0 (mmHg), and carboxyhemoglobin of 37.4% under normobaric oxygen via face mask (10 L/min). He was diagnosed as being in a severe coma due to CO poisoning based on his clinical course. Normobaric oxygen via face mask was continued for two days. He regained consciousness on Day 2 (second day after CO exposure). In brain magnetic resonance (MR) imaging, both fluid-attenuated inversion recovery imaging (FLAIR) and diffusion-weighted imaging (DWI) disclosed symmetric hyperintense lesions in the globus pallidus, which indicated the necrosis of the globus pallidus in the acute phase of CO poisoning (Figure 1A).

**Figure 2:**
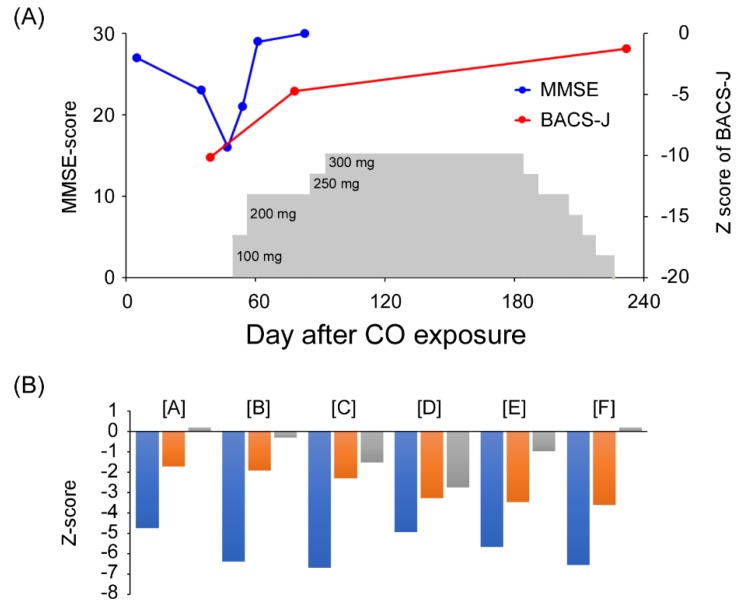
(**A**) Mini-Mental State Examination (MMSE) scores and composite scores of Brief Assessment of Cognition in Schizophrenia—Japanese version (BACS-J) improved by amantadine (AMA) administration. Gray columns indicate the dose of AMA (mg/day). (**B**) Z-scores of subcomponents of BACS-J on Day 39 (blue), Day 78 (orange), and Day 232 (gray). A: verbal memory; B: working memory; C: motor speed; D: verbal fluency; E: attention and processing speed; F: executive function. The z-scores were calculated based on a database of healthy Japanese subjects [5]. Composite scores are the averaged z-scores of the six subcomponents. COP, CO poisoning.

The Mini-Mental State Examination (MMSE) [4] score was 27/30 (Figure 2A) on Day 5; however, on Day 30, he showed gait disturbance, trunk ataxia, and fecal/urine incontinence. His consciousness level was within normal; however, he was confused, with disorientation in terms of time and space. His MMSE score was reduced to 16/30. In addition, the Brief Assessment of Cognition in Schizophrenia—Japanese version (BACS-J) [5] revealed that his cognitive functions were widely impaired (Figure 2). Routine blood laboratory studies were not remarkable. His electroencephalography showed normal background activity (10 Hz). Based on the clinical course and MR images, DNS due to CO poisoning was drastically developing and progressing in this case.

As shown in Figure 1B, both FLAIR and DWI disclosed hyperintense lesions in the subcortical white matter in both hemispheres. Afterward, AMA administration (100 mg/day, per os) was started against Parkinsonism induced by CO poisoning, and the dosage was gradually increased up to the maximal approved dose of 300 mg/day (Figure 2A). About three weeks after AMA administration, typical symptoms including gait disturbance, trunk ataxia, and fecal/urine incontinence had disappeared. Subsequently, memory function improved and the MMSE score on Day 83 was fully recovered, again reaching 30/30, whereas cognitive impairment, including disturbance associated with verbal fluency, attention/processing speed, and executive function, remained to be observed. As for BACS, z-scores of verbal memory and working memory were −1.72 and −1.92, respectively. It took some time for the z-scores of all BACS-J subcomponents to recover (Figure 2B).

On Day 126, FLAIR and DWI showed a decrease in the hyperintense white matter lesions (Figure 1C). In total, he was treated with AMA (50–300 mg/day) for 176 days (Figure 2A). On Day 248, he was discharged from our hospital. He no longer showed recurrence of neurological abnormalities even after AMA administration was stopped about a half year after the CO exposure. On follow-up FLAIR and DWI, the globus pallidus lesions remained; however, the subcortical white matter lesions had disappeared (Figure 1D). Written informed consent was obtained from the patient for the publication of the case report.

## 3. Discussion

Generally, hyperbaric oxygen therapy is considered to be the first-line medication against CO poisoning in patients who have exposure intervals greater than 24 h, loss of consciousness, or higher carboxyhemoglobin concentration [6]. Approximately 70% of patients who survive CO poisoning exhibit various transient symptoms only during the acute phases, and 10% exhibit DNS, representing recurrent neuropsychiatric symptoms occurring after an interval of apparent normality after the apparent resolution of acute symptoms [1,7,8]. Contrary to the acute phase, effective medication for improvement and/or prevention of chronic neuropsychiatric symptoms and DNS is yet to be clarified.

The mechanism of brain damage caused by CO exposure is quite complex [1]. It has been established that brain hypoxia induced by forming carboxyhemoglobin is the major mechanism of various types of brain damage [9]. Hypoxia generates several neurotoxic reactions, including increased glutamatergic transmission and activation of redox reactions [10,11]; however, delay until the occurrence of DNS after improvement of hypoxia cannot be fully explained by hypoxia-induced deficiencies. Therefore, the exact pathomechanism of CO-poisoning-induced DNS is more complex than that of CO-induced hypoxia. Recently, our preclinical study demonstrated that 8 h of CO exposure induced prolonged attenuation of astroglial glutathione synthesis (at least one week). Inhibition of excitatory glutamate receptors, such as NMDA-R and glutamate/α-amino-3-hydroxy-5-methyl-4-isoxazolepropionic acid (AMPA) receptors, prevented the deficits of astroglial glutathione synthesis induced by CO exposure [12]. Furthermore, there is an approved NMDA-R antagonist, AMA-activated system xc-, which is the rate-limiting molecule in the glutathione synthesis pathway [11]. Based on our previous preclinical findings, we administered AMA against Parkinsonism associated with DNS. The first highlight of this case was that the AMA dramatically improved cognitive function as well as Parkinsonism in DNS induced by CO poisoning. Indeed, the follow-up brain MR image on Day 390 showed improvements in hyperintense white matter lesions (Figure 1D). A case study reported that a combined treatment of methylprednisolone and memantine hydrochloride improved Parkinsonism due to CO poisoning [13]. We previously reported that memantine inhibited NMDA-R with activation of system xc- [14], resembling AMA. The preclinical studies could not detect the effects of AMA on dopamine or muscarinic acetylcholine receptor subtypes around therapeutic AMA concentrations [15], whereas clinical studies suggest several types of side effects associated with mild anticholinergic or hyperdopaminergic functions, such as hallucination, dry mouth, and blurred vision [16]. It has been known that anticholinergic agents negatively affect cognitive function [17]. To prevent the dopaminergic and anticholinergic side effects induced by AMA, we gradually decreased the dose of AMA as soon as possible after detecting improvement in scores of the Clinical Global Impressions Severity of Illness scale (CGI-S: 3) on Day 184. Taken together with previous findings, the present case report suggests that multiple pharmacologic targets of AMA (i.e., NMDA-R antagonism and system xc- activation) contribute to recovery from/prevention of DNS induced by CO poisoning.

The second highlight of this case is that the BACS-J was more useful and had higher sensitivity to cognitive impairment induced by DNS compared with MMSE. MMSE has been established as a conventional cognitive impairment test [4]; however, repeated measures analysis using MMSE is affected by learning effects within two months [18]. Contrary to MMSE, BACS-J was originally developed for the assessment of cognition in schizophrenic patients. Recently, clinical research has suggested the effectiveness of BACS-J to assess cognition in patients with bipolar disorder and major depressive disorder as well [19]. In our case, even when the scores of MMSE and two subcomponents of BACS-J—visual memory and working memory—improved, severe deficits of verbal fluency (z-score = −3.28), attention and speed of information processing (z-score = −3.46), and executive function (z-score = −3.6) remained. Subsequently, these also recovered (Figure 2C). We considered that MMSE, generally used in the clinical setting, was not sufficient in this case. Indeed, by retrospective observation [20], the improvement of CGI-S (Day 184) was delayed compared with the MMSE full score (Day 83). Therefore, the discrepancy between MMSE and BACS-J is probably caused by the learning effects. BACS might be an appropriate cognitive test battery in the multidimensional assessment of patients with DNS induced by CO poisoning.

## 4. Conclusions

The present case suggests that AMA is effective against the cognitive impairments and Parkinsonism in DNS induced by CO poisoning. We consider that AMA might become a therapeutic option for DNS due to CO poisoning. In addition, BACS might be a useful test battery to assess cognitive impairment in patients with CO-induced DNS. Further clinical trials are needed to support the present finding.
